# Embedded ethics: a proposal for integrating ethics into the development of medical AI

**DOI:** 10.1186/s12910-022-00746-3

**Published:** 2022-01-26

**Authors:** Stuart McLennan, Amelia Fiske, Daniel Tigard, Ruth Müller, Sami Haddadin, Alena Buyx

**Affiliations:** 1 Institute of History and Ethics in Medicine, Technical University of Munich, Ismaninger Straße 22, 81675 Munich, Germany; 2 Munich Center for Technology in Society, School of Management and School of Life Sciences, Technical University of Munich, Munich, Germany; 3 Munich School of Robotics and Machine Intelligence, Technical University of Munich, Munich, Germany

**Keywords:** Embedded ethics, Artificial intelligence, Medical AI, Technology ethics

## Abstract

The emergence of ethical concerns surrounding artificial intelligence (AI) has led to an explosion of high-level ethical principles being published by a wide range of public and private organizations. However, there is a need to consider how AI developers can be practically assisted to anticipate, identify and address ethical issues regarding AI technologies. This is particularly important in the development of AI intended for healthcare settings, where applications will often interact directly with patients in various states of vulnerability. In this paper, we propose that an ‘embedded ethics’ approach, in which ethicists and developers together address ethical issues via an iterative and continuous process from the outset of development, could be an effective means of integrating robust ethical considerations into the practical development of medical AI.

## Background

Recent advances in computer processing power, the availability of digital ‘big data’, and the development of highly sophisticated algorithms, have created significant opportunities around artificial intelligence (AI) and the sub-fields of machine learning, natural language processing and robotics. AI technologies are already widely used in everyday applications, and it is expected that they will increasingly lead to important changes in society and the economy [1, 2].

Although AI technology promises a number of positive benefits, many important ethical challenges have been identified around AI technology regarding privacy, data protection, transparency and explainability, biases in data, responsibility, and the impact of automation on employment, among other issues [3–5]. These concerns have prompted a rush towards “AI Ethics” to consider how AI technology can be developed and implemented in an ethical manner. A recent scoping review identified 84 documents containing ethical principles or guidelines for AI that have been issued by a wide range of public and private organizations [6]. Assessments of these documents point to an emerging convergence around a set of principles much like the traditional medical ethics principles (e.g. beneficence, non-maleficence, transparency, justice, responsibility) [6–8]. While the classic principles may prove useful in guiding biomedical research and clinical practice, it remains unclear the extent to which overarching high-level principles can help with technical development. Indeed, some have argued that the use of principles alone will be ineffective [9], or worse, that the tech industry’s focus on voluntary ethical compliance is nothing more than a strategic effort to avoid legally enforceable regulation [10, 11].

In the absence of legally enforceable regulations, those developing AI technologies are largely left to translate high-level ethical principles as they see fit [9]. But even where legal regulations have been enacted, it is clear that substantial ethical questions will remain. Consider, by analogy, the role of clinical ethics, where regulations can surely help to guide practitioners’ conduct but moral conundrums are often left open and still call for careful ethical consideration. Although there may be a genuine willingness by tech companies to consider the ethical challenges around AI applications [12], many AI developers do not have the necessary competency to translate unfamiliar high-level principles. Developers come from varied disciplines and professional backgrounds that do not include systematic ethics training. At the same time, few trained ethicists currently work in tech companies, and there is no established culture of practical exchange between these fields. Partly in response to this divergence of fields, there has been efforts to improve the general ethical awareness of those on the technical side of development processes. For example, Floridi and Strait have noted that an increasing number of technology firms are implementing various forms of “ethical foresight analysis” to predict potential ethical issues and the consequences of specific technologies [13]. Further, several leading universities and research institutions now include ethics in their technical curricula with the explicit purpose of raising ethical awareness and capacities for critical reasoning in developers, programmers and engineers [14, 15]. Nevertheless, while these are important steps forward, full proficiency in specifying and applying ethical principles to a wide range of real-world ethical issues requires intensive study and training, as well as a broad toolbox of methodological approaches. Unless educational curricula and corporate trainings are significantly transformed so as to prepare individuals to be experts both in their technical domains and in ethics, it is unrealistic to expect that educational changes will enable all developers to adequately consider the ethical issues arising from the technologies they are developing.

### The need for better integration of ethics into medical AI development

Governments around the world are still grappling with how to regulate AI technologies [16], and it is necessary to consider how AI developers can be practically assisted to identify ethical issues regarding AI technology and reason through how to address these issues. To be sure, the need to assist developers with identifying and addressing ethical aspects can reasonably be seen as spanning across various domains of application, from everyday smartphone apps to self-driving cars and autonomous weapons. However, given the transformative power of AI in medicine and the high-stakes nature of healthcare settings, as we explain, we find it particularly important to work toward the integration of ethics in the development of medical AI. Nonetheless, the broader concern for ethics in technology development, as well as the framework we outline here, should be seen as widely applicable.

The use of AI is projected to transform healthcare [17, 18]. With the ability to learn from large sets of clinical data, medical AI applications have the potential to support a wide range of activities, including diagnosis, clinical decision-making, personalized medicine, clinical research, drug development and administrative processes [19–24]. Additionally, some medical AI applications employ “embodied AI” that are responsive to the patient and their environment through a physically embodied presence, such as artificially intelligent robotic agents or smart prostheses [25–28]. It is expected such medical AI applications will help improve the quality and effectiveness of care, control expenditure, reach underserved or vulnerable populations, and relieve overstretched healthcare services [29–31].

However, in healthcare settings, where medical AI applications may be interacting directly with patients in various states of vulnerability, including reduced well-being and capacity, this technology has a “tremendous capability to threaten patient preference, safety, and privacy” [32]. Indeed, medical AI applications have been found to sometimes be designed without any explicit ethical considerations, and there is a persistent gap between the development of AI and the successful implementation of these tools into clinical environments [29, 33, 34]. With medical AI applications rapidly being implemented into patient care, patients can end up being made to be “unwitting guinea pigs” because medical AI applications are not always tested as rigorously as other medical devices [35]. This situation has already led to serious mistakes, such as an algorithm affecting millions of patients that was found to exhibit significant racial bias [36]. Decisions made during the design and development processes fundamentally determine the final form and operability of the product. Accordingly, it is necessary for robust consideration of ethical issues to be included well before clinical testing and deployment. As AI increasingly moves into the clinic, it is likely that a multi-layered approach for translating ethical principles into AI systems will be most successful, including increased support for ‘bottom-up’ AI ethics [1, 9].

We recognize that to a considerable extent the fields of ethics and technology development exhibit distinctive clashes of cultures. For example, where ethics often draws out discussion by way of analogies and thought experiments, development fields tend to make use of more direct, concrete cases. Where ethics is keen to critique theories and methods, tech-minded researchers are very often focused primarily on usable results. And where the practice of ethics frequently takes substantial time to conduct its analyses, technological developments—whether in academic or corporate settings—are typically produced in ways that best minimize time and other expenditures. Considering these clashes and the realization that AI developers will not likely become experts in ethical reasoning, we argue that the recently proposed ‘embedded ethics’ approach could be an important element for better integrating robust ethical considerations into the development of medical AI [37].

## Main text

### Embedded ethics

The term ‘embedded ethics’ has previously been used in relation to incorporating philosophers in computer science and data science courses, and in relation to programming broad ethical principles directly into algorithms in order to make them “ethical” [14, 38, 39]. However, we use “embedded ethics” in a wide sense, namely to refer to the ongoing practice of integrating ethics into the entire development process—here ethics becomes a truly collaborative, interdisciplinary enterprise. In this way, our approach shares key commonalities with earlier socio-technical paradigms—particularly Responsible Research and Innovation (RRI)—but also bears important points of distinction. Below, we make note of some similarities and differences. In the following sections, we outline the fundamental features of embedded ethics, namely its aims, modes of integration, practices, and requisite expertise and training (see Table 1 for a Summary of Guidance).

**Table 1:** Summary of guidance

Domain	Guidance
Aims	1. Embedded ethics should anticipate, identify, and address ethical issues that arise during the process of developing medical AI
	2. Embedded ethics should work collaboratively with the development team to consider and address these issues via an iterative and ongoing process
Integration	3. Embedded ethics should involve regular exchanges, formally or informally, between ethicists and technical members of the team
Practice	4. Theoretical frameworks employed by embedded ethics should be made clear and explicit
	5. Theoretical frameworks and resulting positions should be explained and justified in terms of specific project goals
	6. The decision-making structure within the team should be clearly established at the beginning of the process
	7. Embedded ethics should consider ways that transparency of analyses in the development of the medical AI could be achieved within the restrictions of confidentiality and intellectual property
Expertise and training	8. Embedded ethics calls for expertise in ethical analysis and proficiency in applied settings, as well as a basic understanding of the AI technology in question and its clinical field of deployment
	9. Opportunities should be created, both before and during a project, for participants to acquire the relevant knowledge and skills to do embedded ethics

#### Aims

The overarching aim of an embedded ethics approach is to help develop AI technologies that are ethically and socially responsible, technologies that benefit and do not harm individuals and society. In order to achieve this goal, ethical considerations are integrated into development processes from the beginning, in order to anticipate, identify, and work to address any ethically significant issues that may arise at all phases of development: planning, ethics approval, designing, programming, piloting, testing, and implementation of the technology in question. Further, it is possible that positioning ethicists throughout the development of medical AI will promote cutting-edge scholarship that helps to anticipate, and not simply respond to, the social and ethical frictions that arise with the application of medical AI technologies.

An “ethical issue” covers points of ethical uncertainty and/or disagreement—within the development team, the literature, or wider society—regarding what course of action ought to be pursued or how an ethical concept should be understood in relation to a medical AI technology [40]. Examples of typical ethical issues include: asking for which goals a device is being developed and who will benefit, either directly or indirectly; what potential risks and harms can be envisaged at an early stage and for whom, and how can these be avoided or minimized through changes in hardware or software; ethical issues specific to AI algorithms (such as potential biases in training data, the need for explicability, and so on); issues pertaining to embodiment (such as potential effects of various robotic interfaces and design); and the mapping of potentially long-term social effects of using the technology (such as replacement of human labor versus improvements in safety and efficiency).

Embedded ethics implies collaborative work between ethicists and the development team to consider and address these sorts of issues via an iterative and ongoing process, borrowing from established approaches such as clinical ethics advisory in hospital settings [41], or ethical, legal and social aspects (ELSA) research in biomedical research [42]. Along with the aforementioned issues that can arise in developing medical AI technologies, previously unknown issues will arise, calling for newfound analyses and ad hoc recommendations put forward after close observations and consultations with developers and relevant stakeholders. In this way, embedded ethics aims to establish transparency regarding any uncertainties or disagreements, and brings new perspectives “to the workbench” by offering a variety of ethically defensible options or strategies for how to address highly pertinent concerns.

In its aims, embedded ethics as we describe it here, closely resembles earlier socio-technical frameworks, namely RRI [43–46]. Indeed, we share with RRI and others the worthwhile goal of considering very broadly the possible impacts of emerging technologies, incorporating diverse stakeholder contributions, at early stages of development [47]. Still, several key differences in aims can be briefly noted. First, according to several prominent accounts of RRI, the framework focuses largely on policy and generally the governance of innovation. For example, in a widely cited work, Stilgoe et al. [48] outline the four dimensions of responsible innovation as: anticipation, reflexivity, inclusion and responsiveness. Importantly, on each dimension, it can be seen that improved governance is a central aim of RRI. The authors make clear that the need RRI addresses is for “improved anticipation in governance” and for “institutional reflexivity in governance”, and so on ([48]: pp. 1570–1571). By contrast, the embedded ethics framework we describe will hopefully be mutually influential with policy and governance—for instance, future policy might better enable the funding and deployment of embedded ethics projects, as we clarify below. However, given our aim of working alongside developers, identifying ethical aspects from the bottom up, the embedded ethics model we employ eschews efforts at governing technological development; and indeed, we believe that doing so is necessary for enabling open dialogues and truly collaborative interdisciplinary work.

Second, although RRI is sometimes linked to a general ‘precautionary principle’ surrounding innovation [45], it has also been described as aiming at the acceptance and marketability of emerging technologies. For example, on one popular definition, von Schomberg [49] describes RRI as a process by which “societal actors and innovators become mutually responsive to each other with a view to the (ethical) acceptability, sustainability and societal desirability of the innovation process and its marketable products” ([49]: p. 0.19). Granted, this process may well lead to products that reflect diverse stakeholder input, and which are more responsive to societal values. Still, we find it crucial to separate the integration of ethical awareness and responsiveness from the effort to increase products’ acceptability or marketability. No doubt, concerns for ‘ethics washing’ [50, 51] may arise when these two features are pursued in tandem, as we discuss further below. Moreover, while developers will naturally aim for their results to be accepted and marketable, ethical issues are most effectively (and most appropriately) raised and addressed in the absence of assumptions—particularly on behalf of ethicists—that technology is the best solution to societal needs.

#### Integration

Depending upon the circumstances and available resources, integration of the embedded ethics approach could occur in one of several possible manners. Arguably, the gold standard for embedded ethics integration would be to have an ethicist, or a team of ethicists, as a dedicated member of the team. Integration here would be facilitated by regular exchanges, whether formally or informally, between ethicists and those on the technical side of development. This approach has been, for example, successfully employed in the field of genomics, where the ethicist Jeantine Lunshof has been embedded for a number of years doing ‘collaborative ethics’ at Harvard’s Wyss Institute for Biologically Inspired Engineering. This collaboration has involved, among other things, Lunshof participating in regular lab meetings, co-authoring papers, and providing support in drafting protocols for the lab’s scientific research [52].

In situations where having an ethicist as a dedicated member of the staff is not feasible due to resource constraints, another option would be to have ethicists working elsewhere regularly join a team’s development meetings, whether in person or virtually via online communication formats. However, a general requirement is that embedded ethics should involve the regular exchange between ethicists and other team members from the beginning of development. It is not sufficient for the development team to call on ethicists working elsewhere only when they perceive ethical issues or social conflicts in the development process. There should be regularly scheduled exchanges between the ethicist(s) and other team members to reduce the risk of ethical issues being overlooked or conflicts being glossed over. In particular, development teams—either academic or corporate—should not be calling upon ethicists only after potential harms are identified or as a response to social or legal pressures, since, again, doing so would undermine the authenticity of truly integrating ethical awareness and critical reasoning capacities in developers.

Often, it is likely that only one ethicist would be embedded in the development process. However, it would also be possible, or indeed sometimes necessary, to have a team of embedded ethicists, particularly in the context of larger initiatives involving several organisations. Whatever form is established, however, an initial protocol should be developed regarding how these regular exchanges will be organized—how often, where, when, who—as well as periodic review and reflection on the process to identify areas for improvement.

#### Practice

Embedded ethics should involve the explicit and robust normative analysis of the issues identified throughout the development processes. This includes explaining and clarifying complex ethical issues so as to allow a clearer understanding of them, and using methods of ethical reasoning to justify or challenge a particular position or course of action [40]. However currently there is no standard approach or accepted set of methods in AI ethics. The use of various ethical positions is common in most branches of applied ethics and can help to illuminate a diversity of perspectives. And although we do not think embedded ethics should be prescriptive about the approach taken to ethical analysis of medical AI, it is crucial that certain criteria concerning transparency and justification are met. In particular, embedded ethicists should (1) make clear and explicit the theoretical ethical positions being invoked in a given normative analysis, and (2) explain and justify why the positions are suitable to meet the specific goals of the project [40]. Regardless of the approach taken to ethical analysis, embedded ethics should be a process of clearly articulating the nature of the identified issue and locating the issue within the relevant literature dealing with the same or comparable concerns [40]. While there may not be a large body of literature on some of the newfound issues that arise, it will often be helpful for the team’s normative analyses to be informed by previous discussions in related domains.

The practice of embedding ethics would ideally involve ethicists accompanying the entire development process, from early decision-making in planning, design and programming, to supporting the regulatory pathway where developments proceed to such stages—for example, by fostering meaningful compliance with ethical review boards and guidelines. At each phase, potential ethical issues would be analyzed and discussed in a collaborative manner and solutions would be sought together. For this to work effectively, the decision-making structure within the team, along with responsibility, must be clearly established from the beginning. This could potentially follow the clinical ethics model where responsibility for development decisions ultimately remains with the lead developer, or it could be developed into a more horizontally distributed decision-making structure [37]. However, given the large amounts of financial investments in AI healthcare technology, embedded ethicists will often be working in contexts with large power differentials. With such a decision-making hierarchy there is a danger that vital decisions will be made without a thorough consideration of ethical aspects, or in a way that allows decision-makers to shirk responsibility. Conversely, however, there is concern in the industry that ethics may lead to development being stymied. To pre-empt challenges in decision-making and other potential tensions between the different fields involved, a process to handle disagreements between developers and ethicists should be developed.

Just like their other colleagues in the development team, embedded ethicists will need to respect confidentiality and intellectual property protection rules. This may pose a tension when considering that embedded ethics seeks to promote transparency in decision-making and ethical analyses. However, an embedded ethics approach should consider ways that a transparent reporting on the analytic process used in the development of the AI healthcare technology could be achieved within these restrictions. Ideally, this would explicitly note the key ethical issues identified and addressed in the development of the technology, the theoretical position used and the reasoning process that led to the pursued course of action, relevant disagreements, and any unresolved issues. Such reporting would be useful not only in developing the embedded ethics approach further, but also for the entire tech field to learn from and debate in relation to future developments through the creation of more embedded ethics literature and case studies.

#### Expertise and training

Embedded ethicists integrated into medical AI development should possess expertise in ethical analysis. This could include not only graduates from specific ethics programs, but also researchers who have been trained in social science and humanities fields that focus on the analysis of ethical issues in science, technology and medicine (such as medical ethics, science and technology studies, sociology, anthropology, or philosophy of science). Embedded ethicists should also have domain-specific understanding and knowledge of the area of technology development in which they will be embedded. Similar to clinical ethicists that have to be able to understand the specificities of a clinical situation, it is important that embedded ethicists have appropriate technology-related knowledge and skills to be able to contribute embedded ethics in a particular AI project. This will often be project specific, however, in the context of AI healthcare technology, it will typically include such things as a basic understanding and knowledge of the general principles of machine learning, robotic design, and the clinical field in which a technology will be deployed. Early pioneers of embedded ethics will likely already possess such “basic understanding and knowledge” from their previous engagements with the field. However, it will be necessary for opportunities to be created, both before and during a project, for participants to be able acquire the relevant knowledge and skills, such as dual-training programs, or internships. This will be particularly important for projects involving junior researchers. In the future it will be necessary to develop training modules geared towards developing such interdisciplinary basic knowledge; such modules could be integrated into currently emerging interdisciplinary classes and curricula at universities and research institutions.

### The merits of embedding ethics in medical AI

While embedded ethics as a collaborative, interdisciplinary approach to AI is in its early stages of growth in academic and industry settings, we believe it is well suited to promote the incorporation of robust ethical considerations into the development of medical AI applications. In this section we want to highlight three key merits of the embedded ethics approach to developing medical AI—namely, the potential to address the uniqueness of medical AI, to mitigate the problem of traditional medical ethics principles, and to remedy the existing regulatory gaps.

#### Addressing uniqueness in medical AI

First, it can be noted that the challenges, and the potential harms, resulting from applications of AI in medicine are unique. AI is unlike other sorts of tools employed in medical practice, largely due to the complex nature of machine learning and artificial neural networks, systems that rely upon hidden or entirely unknown correlations of data for their outputs, yet might take over at least parts of previously expert-driven clinical decision making [53, 54]. As a result, medical practitioners who employ AI technologies, and even the developers themselves, will often have difficulties in predicting a system’s behavior and in explaining to patients why some output was given (such as a diagnosis or preferred treatment recommendation) rather than another. They might also find themselves dealing with issues of shared clinical responsibility with a non-human ‘colleague’. It seems clear, then, that with the introduction of such tools in clinical environments, the very nature of medicine is changing. Notably, changing with it are the crucial relationships between patients and practitioners, and between practitioners and the technical and scientific communities [17, 36]. In this way, we see embedded ethics as a means of continually adapting to our emerging technological environments, helping to assure that AI innovations are developed and deployed in ways that are sensitive to the variety of social and ethical values at stake.

Granted, we do not see embedded ethics as an all-encompassing solution to these layers of challenges, and a multitude of approaches is likely needed, as we return to below. Still, considering the importance of regular exchanges between ethicists and developers working toward medical AI technologies, we are optimistic that the embedded ethics approach will help developers to better see and understand the ethical, social, and political dimensions of their work. Ethicists with experience in applications to medicine and technology can work closely with AI developers, facilitating communication with practitioners, patients, and other stakeholders, in order to help convey the values and demands of healthcare settings (such as transparency, patient autonomy, and fair allocations of resources). Together, embedded ethicists and developers working on medical AI will be in stronger positions to recognize the ethical culture already present in many clinical communities, and can be better equipped to adjust their products accordingly—say, with tailored value-sensitive designs [55]. We are confident that this would represent a significant step forward in terms of producing medical AI that is sensitive to ethical concerns.

#### Mitigating the problem with principles

At present one of the prevailing approaches to AI ethics is the application of traditional ethical principles, often those that are commonly invoked in medical ethics, such as promoting autonomy, non-maleficence, beneficence and justice in caregiving [56]. Yet, it is not clear that the recycling of these undoubtedly valuable principles will be the most effective means of implementing ethics in AI. For example, as Mittelstadt has argued, AI development lacks “(1) common aims and fiduciary duties, (2) professional history and norms, (3) proven methods to translate principles into practice, and (4) robust legal and professional accountability mechanisms” [9]. For these reasons, the classic principles of medical ethics are unlikely to help with AI development, and companies promoting such principles may be doing so in order to avoid legal regulation. Further, we noted that even where AI developers are genuinely willing to address challenges raised by AI, they often simply lack the resources and training to do so effectively.

Here again, it appears that the embedded ethics approach shows great promise in working to resolve the existing challenges. Mittelstadt appropriately draws crucial differences between medical practice and the development of AI. However, the problem with traditional principles becomes less severe when the domain of application is narrowed to a focus on AI in medicine, and when the perspectives and values of medical communities can be fed directly into development at early stages. That is, AI development in general clearly lacks the common aims and history that we find in medical practice. Yet, with developers working together with embedded ethicists toward applications of AI in medicine, the common goals, the history and commonly held values, can thereby be made much clearer and thus more attainable. In this way, traditional ethical principles can at least serve as a guide or starting point to be adapted appropriately and incorporated throughout a project’s development phases. However, like the critics of principlism, we do not assume that the classic principles will apply neatly or consistently when implementing ethics in AI, even for medical applications. As noted above, AI is raising novel challenges and will continue to call for novel resolutions and clear specification of norms [57]. We believe that embedded ethicists can help AI developers to build competencies for identifying and addressing ethical considerations. Accordingly, ethical principles and reasoning generally can be better adapted and more effectively harnessed directly in the development of medical AI.

#### Remedying regulatory gaps

A final merit of the embedded ethics approach starts from the recognition, much like the concerns above, that there is currently little specific regulation for the development and use of AI in medicine. Medical AI technologies are often not tested as rigorously as other products, and although ethics guidelines are rapidly being deployed, the focus is often on the operation of such technologies and thereby too late to profoundly impact its development [58]. Regularly, applications only get assessed when tested in clinical trials and assessed by ethics committees, at which point significant parts of the development have already finished. It is encouraging to see progress in articulating modes of transparency and explainability in AI [54, 59], and in pressing for stronger protections in existing data regulations (such as the European Union´s (EU) General Data Protection Regulation) [60, 61]. However, it is safe to suppose that fully effective regulation of medical AI remains forthcoming.

To help remedy the existing regulatory gaps, and to help supplement newfound regulations, such as the EU’s recent *Artificial Intelligence Act* [62], we find the embedded ethics approach to be particularly well suited to make an efficient and meaningful impact. In an effort to meet the ethical, social and political demands of the digital age, university programs are already increasing in their interdisciplinary research and curricula. Similarly, corporations at the forefront of AI development, as noted, show a willingness to truly engage in ethical practices and are increasingly implementing ethical foresight [12, 13]. Considering these significant advances, it appears that opportunities are arising for creating the necessary modes of training for embedded ethicists, and in placing them where ethics is most needed, namely in the heart of development teams working toward near-future applications of AI. Implementing the embedded ethics approach can, in this way, serve as a swift response to the widespread need for serious consideration of ethics, particularly in the development of medical AI technologies.

## Conclusions

Medical AI offers great promise for improving care, decreasing expenditures, and reaching underserved populations. However, the growing field of medical AI is extensive and applications are far ranging; some cutting-edge applications never reach clinical application due to regulatory concerns, while others move from bench to bedside quickly and with unresolved or unanticipated ethical or social concerns with their use. While various suggestions have emerged from ethicists as well as practitioners at technology firms and research labs, as of yet there is no cohesive approach to address these concerns in order to more fully capitalize on the potential of the field.

In order for medical AI applications to meet its goals, there must be a more systematic process for addressing and anticipating ethical concerns as they arise before products are in clinical trials or in clinical use. While we believe the embedded ethics approach could most easily be implemented in academic institutions and as part of public–private development, it is suited to many different settings, including industry development of medical AI technology and applications. Doing so will help to enable medical AI to realize its potential to transform medicine for the better, in an equitable and safe fashion.

The development of the embedded ethics approach is one step amongst many that will be necessary to tackle the critical ethical, social and political issues that are emerging with the burgeoning application of medical AI. Importantly, the embedded ethics approach can be combined with other specific methodologies such as ethical forecast analysis, as well as with existing proposals in universities for training more AI developers and engineers. Concrete laws and regulations can provide important governance for tech companies and research labs, and ‘softer’ approaches such as AI ethics ‘pledges’ can harness community-level commitments to develop AI only for pro-social intentions [63].The advantage of embedded ethics, while working in conjunction with these various initiatives, is the establishment of a more systematic, integrated, and iterative approach to ethics in AI healthcare innovation. All of these approaches will be necessary as AI becomes an increasingly common-place element of our daily lives and health. However, one of the clear benefits of embedded ethics in relation to existing calls is that it is more systematic, has a broader scope of application, and that it could begin immediately. Highly fluid, embedded ethics can work in a variety of settings, and can be adapted further in light of the specific needs of a development team, product, or process.

Nonetheless, several unresolved issues remain with this proposal. First, even within publicly funded research settings, AI development primarily happens in a highly competitive environment which values efficiency and speed and, in more commercial settings, also profit. Ethical considerations might be ignored when they conflict directly with commercial incentives, and no doubt, ethicists and developers are bound to disagree on numerous substantive issues—consider the tension between transparency and intellectual property. As Metcalf and colleagues have noted, the process of taking ethical considerations seriously is often in tension with industry agendas, and runs the risk of being absorbed into broader corporate commitments to meritocracy, technological solutionism, and market fundamentalism [12]. Ethicists will sometimes work in contexts with extreme power differentials, particularly where corporate or financial interests are involved, as seen in the recent case of Timnit Gebru’s departure from Google. At times, it is likely that some form of enforcement measures will prove necessary, whether through hard regulation, certification, or voluntary measures, in order to counter any tendency for embedded ethics to become merely a form of “ethics washing” or ethical lip service to industry [50]. There are examples from other industries of “ethics seals”, certificates, and compliance programs that could potentially be borrowed and applied in embedded ethics. In our view, it is essential that ethics not serve as a new form of ‘industry self-regulation,’ but rather as an integral part of technological development for healthcare [64].

Secondly, it remains undetermined how embedded ethicists would be paid for their work. We can imagine the possibility for initial public funding to pilot programs within academic research. In order for embedded ethics to be deployed in commercial medical AI development, however, it is possible that there may be industry push-back to funding such programs in the beginning. However, the hiring of ethicists by major tech companies already indicates that company buy-in may not prove to be a significant hurdle [12]. Given the many existing ethics ‘scandals’ that have emerged in relation to the use of AI technologies, it is likely that there is also a strong financial incentive to preventing the development of poorly-informed technologies that have the real possibility to cause harm. Thus, there could be paths for our proposal to be also adopted successfully in industry settings, once the value that embedded ethics brings to the development process and to the bottom line has been established.

Third, there is a clear need for more training for both ethicists and developers and engineers in order to facilitate the kinds of exchange that will be necessary for embedded ethics to work. While existing proposals at leading universities are being developed, it is likely that other models for this training—in particular for professionals that are already working in the field—will prove necessary. Additionally, training, particularly in interdisciplinary and multi-cultural settings, could help to raise awareness of biases, on behalf of both the ethicists and developers involved. By fostering awareness of biases and an environment where diverse perspectives can be openly discussed, we envision the embedded ethics approach working to combat any potentially harmful influences of individual biases concerning the technology in development.

Finally, in order for embedded ethics to succeed, it is necessary to develop clear standards of practice. An established methodological process will help to establish embedded ethics as a distinct community of practice with referenceable standards, case studies, and theoretical infrastructure. This will prove beneficial for all those involved in medical AI, including individuals involved in the creation of training programs, those already working in the medical AI field, ethicists trained in other areas looking to transition to medical AI, as well as other researchers, ethicists and concerned members of the public engaged with the social, ethical and political issues surrounding the use of AI in healthcare.

## Data Availability

Not applicable.

## Abbreviations

AI: Artificial intelligence
ELSA: Ethical, legal and social aspects research
EU: European Union
RRI: Responsible research and innovation
